# Structural dataset from microsecond-long simulations of yeast mitofusin Fzo1 in the context of membrane docking

**DOI:** 10.1016/j.dib.2019.104460

**Published:** 2019-08-31

**Authors:** Astrid Brandner, Dario De Vecchis, Marc Baaden, Mickael M. Cohen, Antoine Taly

**Affiliations:** a Institut de Biologie Physico-Chimique-Fondation Edmond de Rothschild, PSL Research University, Paris, France; b CNRS, Université de Paris, UPR 9080, Laboratoire de Biochimie Théorique, 13 rue Pierre et Marie Curie, F-75005, Paris, France; c Laboratoire de Biologie Cellulaire et Moléculaire des Eucaryotes, Sorbonne Université, CNRS, 16 UMR 8226, Paris, France

**Keywords:** Mitofusin, Fzo1, Mitochondrial fusion, Tethering, Structure, Molecular dynamics, Modelling

## Abstract

In this work we present a novel set of possible auto-oligomerisation states of yeast protein Fzo1 in the context of membrane docking. The dataset reports atomistic models and trajectories derived from a molecular dynamics study of the yeast mitofusin Fzo1, residues 101–855. The initial modelling was followed by coarse-grained molecular dynamics simulation to evaluate the stability and the dynamics of each structural model in a solvated membrane environment. Simulations were run for 1 μs and collected with GROMACS v5.0.4 using the martini v2.1 force field. For each structural model, the dataset comprises the production phase under semi-isotropic condition at 1 bar, 310 K and 150 mn NaCl. The integration step is 20 fs and coordinates have been saved every 1 ns. Each trajectory is associated with a ready-available visualization state for the VMD software. These structural detailed informations are a ready-available platform to plan integrative studies on the mitofusin Fzo1 and will aid the community to further elucidate the mitochondrial tethering process during membrane fusion. This dataset is based on the publication “Physics-based oligomeric models of the yeast mitofusin Fzo1 at the molecular scale in the context of membrane docking.” (Brandner and De Vecchis et al., 2019)”.

**Table:** Specifications Table

Subject	Structural Biology
Specific subject area	Molecular modelling and molecular dynamics simulation
Type of data	Structural Data: atomic coordinates in PDB format, trajectory files from molecular dynamics simulations in XTC format Visualization file: vmd visualization state
How data were acquired	PDB coordinates of the initial structural models were taken as starting point from a previously published Fzo1 model [2]. Models were assembled using the MODELLER [3] and UCSF Chimera [4] software packages. Trajectories were obtained from molecular dynamics simulations using GROMACS v5.0.4 [5] and the martini v2.1 force field [6], [7]. The production runs were 1 μs long. VMD states were created using VMD v.1.9.3 [16]
Data format	Raw
Parameters for data collection	Molecular dynamics simulations of 1 μs length were run semi-isotropically at 1 bar, 310 K, with a NaCl concentration of 150 mM. The integration step was 20 fs.
Description of data collection	The trajectories reported here were obtained from a 1μs-long production run. Frames were saved every 1 ns. Visualization states were acquired using VMD v1.9.3
Data source location	Institution: Laboratoire de Biochimie Theorique - UPR 9080, Institut de Biologie Physico-Chimique City/Town/Region: 13 rue Pierre et Marie Curie, Paris Country: France
Data accessibility	Repository name: Mendeley Data Data identification number: 10.17632/xbkftgt3gg.1 Direct URL to data:https://data.mendeley.com/datasets/xbkftgt3gg/draft?a=d3e4e97d-e270-4375-b93c-853cf2f4e804
Related research article	Astrid Brandner, Dario De Vecchis, Marc Baaden, Mickael M. Cohen and Antoine Taly Physics-based oligomeric models of the yeast mitofusin Fzo1 at molecular scale in the context of membrane docking. Mitochondrion DOI:Manuscript number: MITOCH_2019_45

**Table:** 

**Value of the Data** • Our data provide atomistic detailed Fzo1 oligomers based on a structural model which has been previously validated experimentally. The oligomers represent cis and trans assemblies in the context of mitochondrial membrane tethering. These models allow for instance to derive experimentally testable hypotheses. • Sharing our data will widely contribute to stimulate the community that work on mitochondrial fusion and dynamics. Experimentalists such as crystallographers and structural biologists will benefit from our structural models. Similarly, computational biologists and biophysicists will take advantage from our datasets which is represented by atomistic coordinates. • Our data is represented by atomistic coordinates that can be used to precisely plan in vivo and in vitro experiments on mitofusins. Having structural data will allow researchers to sustain further our model validation and also to advance the elucidation of the mitochondrial tethering process. In addition, the coordinates can be used in future dynamics studies in conjunction with other actors involved in membrane fusion, once they will become available. • These data represent the first step towards a rigorous dynamic assessment of the mitofusin Fzo1 oligomers in a membrane environment and in the context of membrane tethering. There are currently no other full models of the mitofusins available.

## 1 Data

Trajectories are classified in two categories: dimers and tetramers. Three different cis-dimers are presented: wide and narrow head-to-head complexes, where two Fzo1 molecules interact via their GTPase domain; and a back-to-back complex for the closed model where two Fzo1 molecules interact through their heptad repeats (HR) domains. For the tetramers, we also present three possible oligomerisation structures: in the first, the Fzo1 monomers are in open conformations and interacting through their GTPase domains; in the second, the cis interaction occurs via the HRs oriented in a parallel fashion and the trans interaction occurs via the GTPase domain and third, a Fzo1 trans-tetramer in closed conformation where the cis interaction occurs towards the GTPase domain and the trans interaction via the respective HR domains oriented in an antiparallel fashion. All the files are summarised in Table 1.

**Table 1:** List of shared files with descriptions ordered by olgimerisation type and origin of coordinates.

System type	Origin of coordinates	Filename	File description
Cis-dimers	Models	CisBack_Model.pdb	PDB coordinates for the initial atomistic cis back-to-back dimer model of Fzo1.
		CisHeadNarrow_Model.pdb	PDB coordinates for the initial atomistic cis head-to-head narrow dimer model of Fzo1
		CisHeadWide_Model.pdb	PDB coordinates for the initial atomistic cis head-to-head wide dimer model of Fzo1.
	Coarse-grained molecular dynamics	CisBack.pdb,	PDB coordinates for the initial structure from the coarse-grained molecular dynamics simulation, full production run trajectory and visualization file of the cis back-to-back dimer of Fzo1.
		CisBack.xtc,	
		state_CisBack.vmd	
		CisHeadNarrow.pdb,	PDB coordinates for the initial structure from the coarse-grained molecular dynamics simulation, full production run trajectory and visualization file of the cis head-to-head narrow dimer of Fzo1.
		CisHeadNarrow.xtc,	
		state_CisHeadNarrow.vmd	
		CisHeadWide.pdb,	PDB oordinates for the initial structure from the coarse-grained molecular dynamics simulation, full production run trajectory and visualization file of the cis head-to-head wide dimer of Fzo1
		CisHeadWide.xtc,	
		state_CisHeadWide.vmd	
Tetramers	Models	TetramerBackAntiparallel_Model.pdb	PDB coordinates for the initial atomistic model of the cis head-to-head, trans back-to-back antiparallel interaction of Fzo1.
		TetramerBackParallel_Model.pdb	PDB coordinates for the initial atomistic model of the cis back-to-back parallel, trans head-to-head interaction of Fzo1.
		TetramerHeadWide_Model.pdb	PDB coordinates for the initial atomistic model of the cis head-to-head wide, trans head-to-head interaction of Fzo1
	Coarse-grained molecular dynamics	TetramerBackAntiparallel.pdb TetramerBackAntiparallel.xtc state_TetramerBackAntiparallel.vmd	PDB coordinates for the initial structure from the coarse-grained molecular dynamics simulation, full production run trajectory and visualization file of the cis head-to-head, trans back-to-back antiparallel interaction of Fzo1
		TetramerBackParallel.pdb TetramerBackParallel.xtc state_TetramerBackParallel.vmd	PDB coordinates for the initial structure from the coarse-grained molecular dynamics simulation, full production run trajectory and visualization file of the cis back-to-back parallel, trans head-to-head interaction of Fzo1.
		TetramerHeadWide.pdb TetramerHeadWide.xtc state_TetramerHeadWide.vmd	PDB coordinates for the initial structure from the coarse-grained molecular dynamics simulation, full production run trajectory and visualization file of the cis back-to-back parallel, trans head-to-head interaction of Fzo1.of the cis head-to-head wide, trans head-to-head interaction of Fzo1.

## 2 Experimental design, materials, and methods

The structural models presented here build upon an experimentally validated model of the monomeric unit, namely our previously published model of Fzo1 in a closed conformation [2]. It is first used to generate a monomeric model of the open conformation. Those two models are the basis for the construction of Fzo1 dimer models in *cis* and then tetramers via the dimerization in *trans* of Fzo1 *cis*-dimers.

First, we generate the Fzo1 GTPase dimer construct. Two chains of the Fzo1 model in closed conformation [2] were placed by superimposing their GTPase domains onto that of BDLP in open conformation [8]. Then, only the coordinates of the two fragments that comprise the Fzo1 GTPase domain (residues 188–461) were retained to form the final GTPase domain dimer model. The loop refinement tool implemented in MODELLER [3] was used to remove a clash in both chains involving an unresolved loop in the template 2J68 [9] (residues 215–219). Models were ranked according to the discrete optimized protein energy (DOPE) method [10], selecting the best-scoring loop out of 20 candidates.

### 2.1 Cis-dimer structural models

#### 2.1.1 CisHeadNarrow_Model

Two Fzo1 chains in closed conformation [2] were oriented facing each other within a compatible distance to accommodate two interacting GTPase domains. Subsequently, the coordinates of residues 188–440 enclosed between hinges 2a and 2b (i.e., comprising the GTPase domain) were removed from both chains and replaced with the GTPase dimer construct described above. The latter was manually positioned between the two deleted chains, resulting in the head-to-head interaction dimer. The backbone interruptions were connected using the loop refinement tool implemented in MODELLER [3] using positions 185–188 and 436–445 as anchors. Solutions were ranked according to the DOPE method [10], selecting the best-scoring loop out of 20 candidates.

#### 2.1.2 CisBack_Model

Two chains of the Fzo1 model in closed conformation [2] were manually oriented with respect to each other to generate the back-to-back interaction. In the resulting model system, the HR domains face each other in a parallel fashion.

#### 2.1.3 CisHeadWide_Model

The coordinates from BDLP in open conformation [8] derived from the electron density map of native BDLP lipid tubes (accession code: EMD-1589) were used as a template to model Fzo1 in open conformation. Starting from our previous Fzo1-BDLP target-template alignment [2], we introduced homologous chain breaks on the Fzo1 model [2], resulting in five rigid blocks. Each fragment was superposed to its corresponding fragment in 2W6D to reconstitute the orientation found in BDLP. The MatchMaker tool from the UCSF Chimera software [4] was used during this procedure. The loop refinement tool implemented in MODELLER [3] enabled us to complete the model in the resulting backbone interruptions and to remove a clash in both chains between the side chain of the Lys271 and the backbone of the Ala401 residues, using positions 268 and 273 as anchors. Solutions were ranked according to the DOPE method [10], selecting the best-scoring loop out of 10 models.

### 2.2 Trans-tetramer structural models

#### 2.2.1 TetramerHeadWide_Model

Two Fzo1 *CisHeadWide* models obtained as described above were manually oriented to mimic the *interactions* in trans towards their respective GTPase domains. In the resulting model system, the two transmembrane segments are located at opposite ends.

#### 2.2.2 TetramerBackAntiparallel_Model

The transmembrane segment of two Fzo1 *CisHeadNarrow* described above were manually oriented at opposite ends to optimize the interaction between their respective HR domains oriented in an antiparallel fashion. Note that this system, although antiparallel, could also be considered as back-to-back.

#### 2.2.3 TetramerBackParallel_Model

Two Fzo1 *CisBack* models obtained as described above were initially positioned with the respective transmembrane segments at opposite ends to mimic the supposed tethering process. Subsequently, the coordinates of residues 101–491 and 816–855 enclosed between hinges 1a and 1b were removed from the two resulting juxtaposing chains. Then the GTPase dimer construct was built. We superposed the GTPase domain alpha carbons of two Fzo1 chains in closed conformation [2] with the human mitofusin dimer [11]. This choice was motivated and directly inspired by the work from Gao and collaborators, who proposed a possible Mfn1 *trans* cross oligomer [11]. From the resulting Fzo1 dimer, only the GTPase domain and the 3-helix bundle were selected and used to replace the aforementioned deleted portions, thus generating the *trans* head-to-head interaction. A clash in one chain (residues 215–218) was removed from the resulting Fzo1 dimer using the loop refinement tool implemented in MODELLER [3]. Models were ranked according to the DOPE method [10], selecting the best-scoring loop out of 10 candidates. The same tool was used to reconstitute the backbone interruptions. Positions 491–495 and 812–816 were selected as anchors. The best-scoring loop was selected out of 10 candidates.

### 2.3 Molecular dynamics simulation protocol

Topologies to run coarse-grained (CG) simulations were generated with the martinize tool choosing the martini v.2.1 force field with an elastic network [6], [7] The initial all atom coordinates were obtained from the published models in the related research paper [1]. The force bond constant was set to 500 kJ mol^−1^ nm^−2^ with lower and upper elastic bond cutoffs of 0.5 and 0.9 nm, respectively. Firstly, 5000 steps of steepest descent with position restraints for the protein were run followed by 5000 steps without restraints. The obtained coordinates were inserted in a POPC:POPE (1:1) membrane via the insane tool [12] where the membrane position was manually set up to match the reported transmembrane regions corresponding to residues 706–726 and 737–757 according to UniProt numbering. All systems were fully solvated to mimic an environment of 150 mM of NaCl solution. The final systems followed the same simulation protocol using the GROMACS 5.0.4 software [5] with periodic boundary conditions. A further 5000 steps of steepest descent minimisation with position restraints of 1000 kJ mol^−1^nm^−2^ in protein and lipids were followed by 5000 steps without position restraints. Equilibration was performed in three stages, with timesteps of 20 fs. Firstly, 25000 steps of equilibration were run at 310 K using the V-rescale thermostat [13] and semi-isotropic pressure coupling via Berendsen barostat [14] with position restraints of 1000 kJ mol^−1^ nm^−2^ for protein and lipids, followed by the same setup without position restraints. Finally, the last equilibration step was run for 50000 steps with the V-rescale thermostat (coupling constant tau_t = 1 ps) and semi-isotropic coupling with Parinello-Rahman barostat [15] (coupling constant tau_p = 12 ps). Production runs were 1 μs long for all six systems, following the same parameters as those used in the last equilibration setup. The trajectories shared in this work correspond to the coordinates of all non-water atoms from the production run for each of the systems. They were centred on the protein to facilitate the visualization. Protein residues have been sequentially numbered, i.e each monomer consists of protein residues 1–755, 756–1510, 1511–2265 and 2266–3020. The martini forcefield naming scheme was retained, where the backbone is represented by one bead only (BB).

#### 2.3.1 Visualization information

To facilitate the visualization of each trajectory, we added a vmd state file to be loaded in VMD [16] alongside the trajectory without any need of extra processing. The colouring was made to highlight the functional domains of the protein following the same scheme as in our original paper [1]. The domains highlighted by colour are: violet, HRN; green, HR1; orange, HR2; red, GTPase and yellow: transmembrane. Phosphorus atoms (blue) from lipid bilayer headgroups are depicted in space-filling representation.
